# Knockdown of the UL-16 binding protein 1 promotes osteoblast differentiation of human mesenchymal stem cells by activating the SMAD2/3 pathway

**DOI:** 10.1186/s12891-024-07341-0

**Published:** 2024-03-13

**Authors:** Zhen Lai, Mingming Li, Xiaodong Yang, Zhenjie Xian

**Affiliations:** 1 Department of Orthopedic Surgery, Huadu District People’s Hospital of Guangzhou, 48 Xinhua Road, Xinhua Street, Huadu District, Guangzhou, 510800 Guangdong China; 2 Shiling Town Health Center, 19 Qiling Street, Huadu District, Guangzhou, 510800 Guangdong China

**Keywords:** Osteoporosis, Osteoblast differentiation, ULBP1, TNF-β, SMAD

## Abstract

Osteoporosis is caused by the imbalance of osteoblasts and osteoclasts. The regulatory mechanisms of differentially expressed genes (DEGs) in pathogenesis of osteoporosis are of significant and needed to be further investigated. GSE100609 dataset downloaded from Gene Expression Omnibus (GEO) database was used to identified DEGs in osteoporosis patients. KEGG analysis was conducted to demonstrate signaling pathways related to enriched genes. Osteoporosis patients and the human mesenchymal stem cells (hMSCs) were obtained for in vivo and in vitro resaerch. Lentivirus construction and viral infection was used to knockdown genes. mRNA expression and protein expression were detected via qRT-PCR and western blot assay separately. Alkaline phosphatase (ALP) activity detection, alizarin Red S (ARS) staining, and expression of bone morphogenetic protein 2 (BMP2), osteocalcin (OCN) and Osterix were evaluated to determine osteoblast differentiation capacity. UL-16 binding protein 1 (ULBP1) gene was upregulated in osteoporosis and downregulated in differentiated hMSCs. Knockdown of ULBP1 increased ALP activity, mineralization ability evaluated by ARS staining, expression of BMP2, OCN and Osterix in differentiated hMSCs. Furthermore, rescue experiment demonstrated that suppressed ULBP1 boosted osteoblast differentiation by activating TNF-β signaling pathway. Knockdown of ULBP1 gene could promoted osteoblast differentiation by activating TNF-β signaling pathway in differentiated hMSCs. ULBP1 may be a the Achilles’ heel of osteoporosis, and suppression of ULBP1 could be a promising treatment for osteoporosis.

**Supplementary Information:**

The online version contains supplementary material available at 10.1186/s12891-024-07341-0.

## Introduction

Osteoporosis is a systemic bone disorder characterized by low bone mass and increased brittleness, which can easily lead to fractures, particularly in older individuals [1, 2]. More than 2,000,000 osteoporosis-related fractures occur each year according to the National Osteoporosis Foundation of America [3]. An imbalance between osteoblasts and osteoclasts in healthy bone tissues is the main cause of low bone mass which directly leads to osteoporosis [4]. Hence, it is of great significance to find the potential molecular mechanisms that maintain the osteoblast-osteoclast balance in bone cells.

Differentially expressed genes (DEGs) which were identified by gene microarray analysis has been reported to play regulatory roles in various diseases [5–7]. The reanalysis of these data and the prediction of targets can help to develop new diagnostic and therapeutic strategies [8]. For instance, 496 and 291 DEGs were identified in osteoporotic rats before and after astragalus polysaccharide (APS) treatment, respectively, indicating that APS may play a role in the treatment of osteoporosis by reversing the expression of 53 DEGs [9]. Similarly, Yu T et al. conducted enrichment and protein-protein interaction network analyses of DEGs identified from three datasets, and found three hub genes that promote osteoporosis [7]. Hence, it is feasible to screen for potential genes associated with osteoporosis and to verify these genes through comprehensive follow-up experimental studies.

Bone mesenchymal stem cells (MSCs), as the main source of osteoblasts, have weakened osteogenic differentiation ability and enhanced adipogenic differentiation ability in patients with osteoporosis [10, 11]. Application of MSCs therapy is one of the promising treatment to renew bone tissue in the body, so the regulation of MSCs osteogenic differentiation will be helpful for the treatment of osteoporosis. The UL-16 binding protein 1 (*ULBP1*) gene is a ligand of natural killer group 2 member D (NKG2D), mediates natural killer (NK) cell cytotoxicity [12, 13]. Earlier bioinformatics analysis results suggested that ULBP1 may be a regulatory DEGs in osteoporosis, which was also verified in this study [14]. Therefore, this study aims to evaluate the role of ULBP1 in osteoblast differentiation and the underlying mechanisms of influencing potential signaling pathways. These findings may help to uncover novel diagnostic biomarkers.

## Materials and methods

### Microarray expression profiling

Microarray profile GSE100609 of osteoporosis related genes of Indian post menopausal females and non-osteoporotic post menopausal females were downloaded from the Gene Expression Omnibus (GEO) database (http://www.ncbi.nlm.nih.gov/geo). All differentially expressed genes (DEGs) of two groups were analyzed by an online analysis program Geo2R provided by GEO database. *P* < 0.05 and |log_2_FC| ≥ 1.5 is the standard of screening DEGs. Pearson correlation was used to analysis the correlation between DEGs, and the KEGG analysis was applied to analyze the related-pathways of enriched genes.

### Patients

44 postmenopausal women diagnosed as osteoporosis in Huadu District People’s Hospital of Guangzhou and 40 healthy postmenopausal women were included in this research under the approval of Ethics Committee of Huadu District People’s Hospital of Guangzhou. Fasting blood (5 mL) was obtained from all subjects who have signed the informed consents,and was centrifuged within 15 min after collection with a relative centrifugal force of 1200×g for 10 min. Total serum RNA was then collected using the Trizol reagent (Invitrogen, CA, USA) under the guidance of the manufacturer.

### Cell culture

The human mesenchymal stem cells (hMSCs) were purchased from American Type Culture Collection (ATCC, MD, USA) and cultured in mesenchymal stem cell basal medium (Gibco, CA, USA) at 37 °C in a 5% CO_2_ incubator.

### Osteogenic inductive medium (OIM) for osteoblast differentiation

The hMSCs at concentration of 2 **×** 10^4^ /well were seeded in 6-well plates (Thermol Fisher, CA, USA) and cultured in original α-MEM medium (Gibco, CA, USA). After 24 h, 10% foetal Bovine Serum (FBS; Gibco, CA, USA), 50 µM l-ascorbic acid (Gibco, CA, USA), 10 mM β-glycerophosphate (Gibco, CA, USA), as well as 100 nM dexamethasone (Gibco, CA, USA) were then added. The medium was changed every another day. The hMSCs before osteogenic differentiation induction were classified as control group, and the hMSCs after osteogenic differentiation were classified as OMI group.

### Alkaline phosphatase (ALP) activity detection

ALP activity was detected to distinguish osteoblasts by determining early mineralization level of differentiated hMSCs [15]. ALP activity was measured by a fluorescence detection kit in accordance with the manufacturer’s protocol (Nanjing Jiancheng Institute, Nanjing, China).

### Alizarin Red S (ARS) staining

ARS staining was conducted to detect the later mineralization state of differentiated hMSCs [15]. Briefly, differentiated hMSCs were fixed in 4% formaldehyde (aladdin, Shanghai, China) for 15 min, then stained with 2% alizarin red solution (Beyotime, Nantong, China) at room temperature. After washed by distilled water, stained hMSCs were observed by a microscope (Dynex Technologies, VA, USA) at 450 nm.

### Lentivirus construction and viral infection

Cell infection was carried out after the hMSCs were successfully differentiated. All plasmids used were purchased from GenePharma Company (Shanghai, China) and were listed below: UL-16 binding protein 1 (ULBP1) short-hairpin RNAs (shRNAs): 5’-GCAGCTTTATAAACAGCCGTG-3’ (1#), 5’-GCCGTGGTGTGAGCCTCGAAG-3’ (2#). mothers against decapentaplegic homolog 2 (SMAD2) shRNA: 5’-GGTGAAGAATTGGAGCCTTAA-3’. In brief, the differentiated hMSCs were infected with sh-NC, sh-ULBP1, or sh-SMAD2 supplemented with 6 µg/mL polybrene (Gibco, CA, USA) for 12 h, and then the transfection medium was replaced. After 72 h, the infected cells were screened with 1 µg/mL purinomycin (Gibco, CA, USA) for subsequent experiments. We used RT-PCR to analyze the transfection efficiency.

### qRT-PCR

The expression of genes were detected including ULBP1, bone morphogenetic protein 2 (BMP2), osteocalcin (OCN), Osterix, inhibin beta A chain (INHBA), repulsive guidance molecule A (RGMA), growth/differentiation factor 5 (GDF5) and SMAD2. After the hMSCs were differentiated or transfected successfully, total RNA was collected by Trizol (Invitrogen, CA, USA) and then transcribed into cDNA via PrimeScript RT reagent kit (TaKaRa, Tokyo, Japan). Quantification of all gene transcripts was performed by qRT-PCR using THUNDERBIRD SYBR® qPCR Mix (Toyobo life science, Osaka, Japan). The relative expressions of genes were calculated using the 2^−ΔΔCt^ method. The primer sequences were shown in Table 1.

**Table 1:** Primer sequences

Gene name	Forward primer (5’-3’)	Reverse primer (5’-3’)
*ULBP1*	TAAGTCCAGACCTGAACCACA	TCCACCACGTCTCTTAGTGTT
*BMP2*	ACCCGCTGTCTTCTAGCGT	TTTCAGGCCGAACATGCTGAG
OCN	CACTCCTCGCCCTATTGGC	CCCTCCTGCTTGGACACAAAG
Osterix	CCTCTGCGGGACTCAACAAC	AGCCCATTAGTGCTTGTAAAGG
*INHBA*	CCTCCCAAAGGATGTACCCAA	CTCTATCTCCACATACCCGTTCT
*RGMA*	CCTCAGGACTTTCACCGACC	CGTTCTTAGAGCCATCCACGAA
*GDF5*	GCTGGGAGGTGTTCGACATC	CACGGTCTTATCGTCCTGGC
*SMAD2*	CGTCCATCTTGCCATTCACG	CTCAAGCTCATCTAATCGTCCTG

### Western blot assay

hMSCs of each group were lysed to to obtain total protein extracted by RIPA reagent (Sigma, NJ, USA). Protein quantification was carried out by a BCA protein assay (Thermol Fisher, CA, USA). Subsequently, proteins were isolate by 10% SDS-PAGE and electrotransferred to polyvinylidene fluoride membranes (Millipore, MA, USA). Afterwards, the membranes were blocked before incubation by primary antibodies including anti-BMP2 (ab214821, 1/1000), anti-OCN (ab133612, 1/1000), anti-Sp7/Osterix (ab227820, 1/1000), anti-SMAD2 (ab40855, 1/2000), anti-SMAD3 (ab40854, 1/2000), anti-p-SMAD2 (ab280888, 1/1000), and anti-p-SMAD3 (ab52903, 1/2000) overnight at 4 °C. Then the membranes were washed by Tris buffered saline with Tween 20 (Gibco, CA, USA), followed by secondary antibody (ab205718; 1/10,000) for 2 h. Finally, the protein band grayscale value was analyzed by Image Lab software (Bio-Rad, Hercules, USA).

### Ovariectomized (OVX) mouse model of osteoporosis establishment

Female C57BL/6 mice (10 weeks of age) were randomly divided into 4 groups with 6 mice per group. Ovariectomy or sham surgery is performed under general anesthesia. Both ovaries were removed under sterile conditions. In the sham group, only part of the adipose tissue around the ovaries was removed. Lentivirus intramedullary injection was given 4 weeks after ovariectomy. Specifically, a 5-mm longitudinal incision was made along the medial side of the quadriceps femoris patella complex. Lateral dislocation of the patella was performed to expose the intercondylar sulcus. A fine Kirschner wire was drilled in, a 26-gauge needle was inserted, and 15 µL of sh-ULBP1 lentivirus (5 × 10^7^ /mL) was injected into the medullary cavity. The quadriceps femoris patella complex was then resutured. All mice were anesthetized with sodium pentobarbital (P276000, 100 mg/kg, i.p. AmyJet Scientific, Wuhan, China) two months later, and the femurs were removed after the last injection.

### Bone loss evaluation

A Scanco vivaCT 40 (Scanco Medical AG, Bassersdorf, Switzerland) was employed for micro-computed tomography (µCT). Using 6-µm pixel size, we set an X-ray source at 60 kV and scanned the excised left distal metaphysis of the femurs. We focused on a ~ 0.5 mm proximal region in the most distal part of the growth plate. The femurs of mice were fixed with 4% paraformaldehyde for 24 h and then scanned by a Scanco vivaCT 40 (Scanco Medical AG, Bassersdorf, Switzerland). An X-ray source at 60 kV was set, the femurs were then scanned. Several bone-related parameters were analyzed, including the trabecular separation (Tb.Sp, mm), trabecular thickness (Tb.Th, µm), bone volume/total volume (BV/TV, %), bone surface-to-volume ratio (BS/BV, mm^− 1^), and trabecular number (Tb.N, mm^− 1^). For bone mineral density (BMD, g/cm^3^) determination, Brukermicro-CT BMD calibration phantoms were used with calcium hydroxyapatite concentrations of 0.25 and 0.75 g/cm^3^.

### Statistical analysis

Statistical analyses were performed using the GraphPad Prism8.3 software (GraphPad, San Diego, USA). Each experiment was performed at least 3 times. All data were expressed as mean ± SD, and Student’s t-test was used to analyze the differences between the two groups while one-way ANOVA was for multiple groups analysis. Meanwhile, Turkey test was used to verify ANOVA for pairwise comparisons. Pearson correlation was used to analysis the correlation between expression of DEGs. *P* < 0.05 was regarded as statistically significant.

## Results

### ULBP1 is an aberrant expressed gene in osteoporosis

**Fig. 1:**
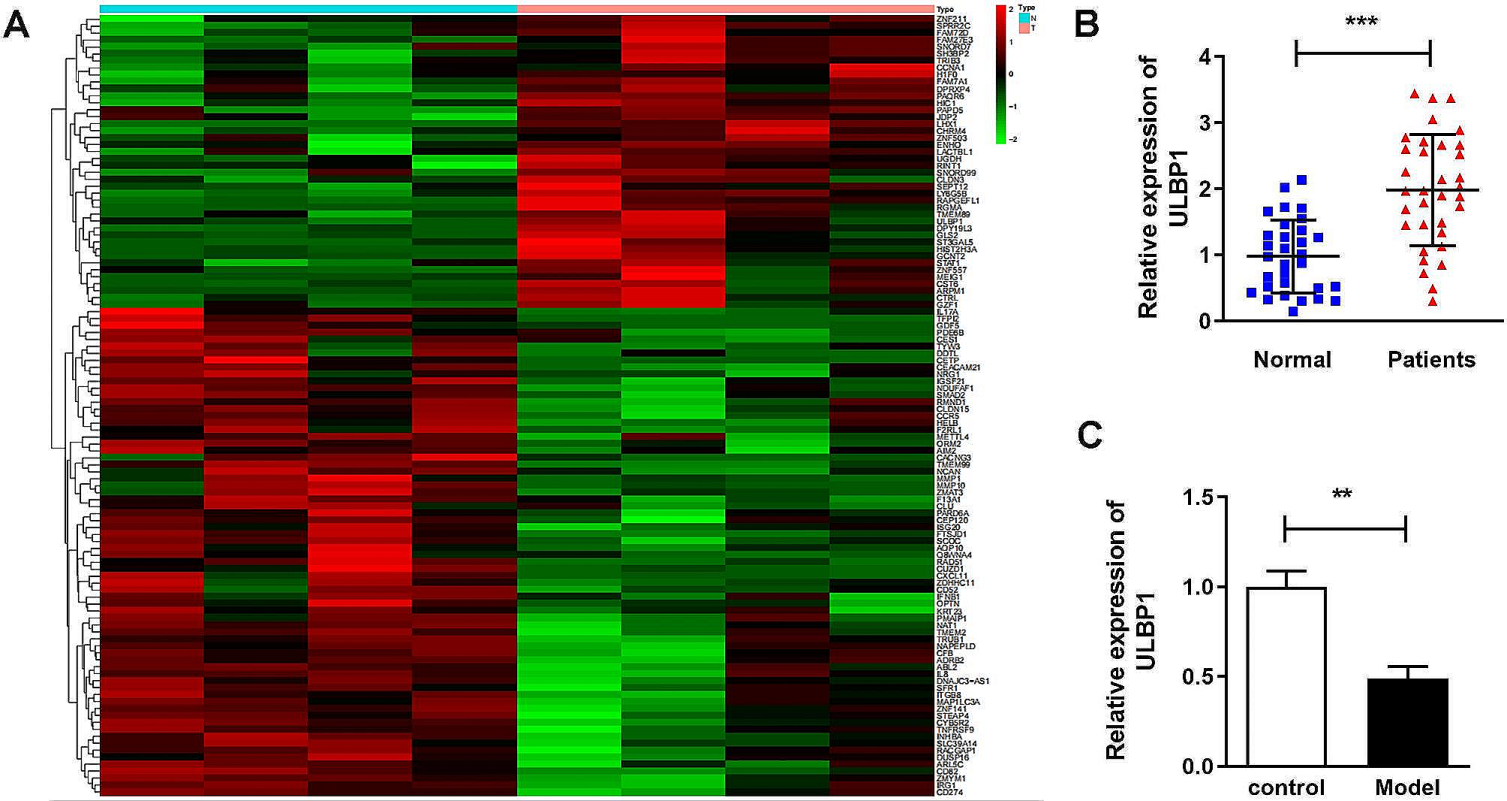
ULBP1 is an aberrantly expressed gene in osteoporosis. Heat map of DEGs in osteoporosis identified using the GSE100609 dataset. B. mRNA expression of ULBP1 was examined using PCR in the serum of patients with osteoporosis and compared to that in healthy controls. C. mRNA expression of ULBP1 in hMSCs before and after differentiation was examined using PCR. **P < 0.01, ***P < 0.001

After preliminary screening, 112 DEGs were identified in osteoclast precursor cells sorted from circulating monocytes of osteoporotic patient s and normal controls, among which *ULBP1* gene was significantly up-regulated (Fig. 1A). In addition, mRNA expression of ULBP1 was also over-expressed in serum of osteoporosis patients whereas suppressed in osteogenic differentiated hMSCs (Fig. 1B and C). The protein levels of ULBP1 were also found to be elevated in serum samples of patients with osteoporosis (Fig. S1).

### Differentiated hMSCs reveals the osteoblast differentiation capacity

**Fig. 2:**
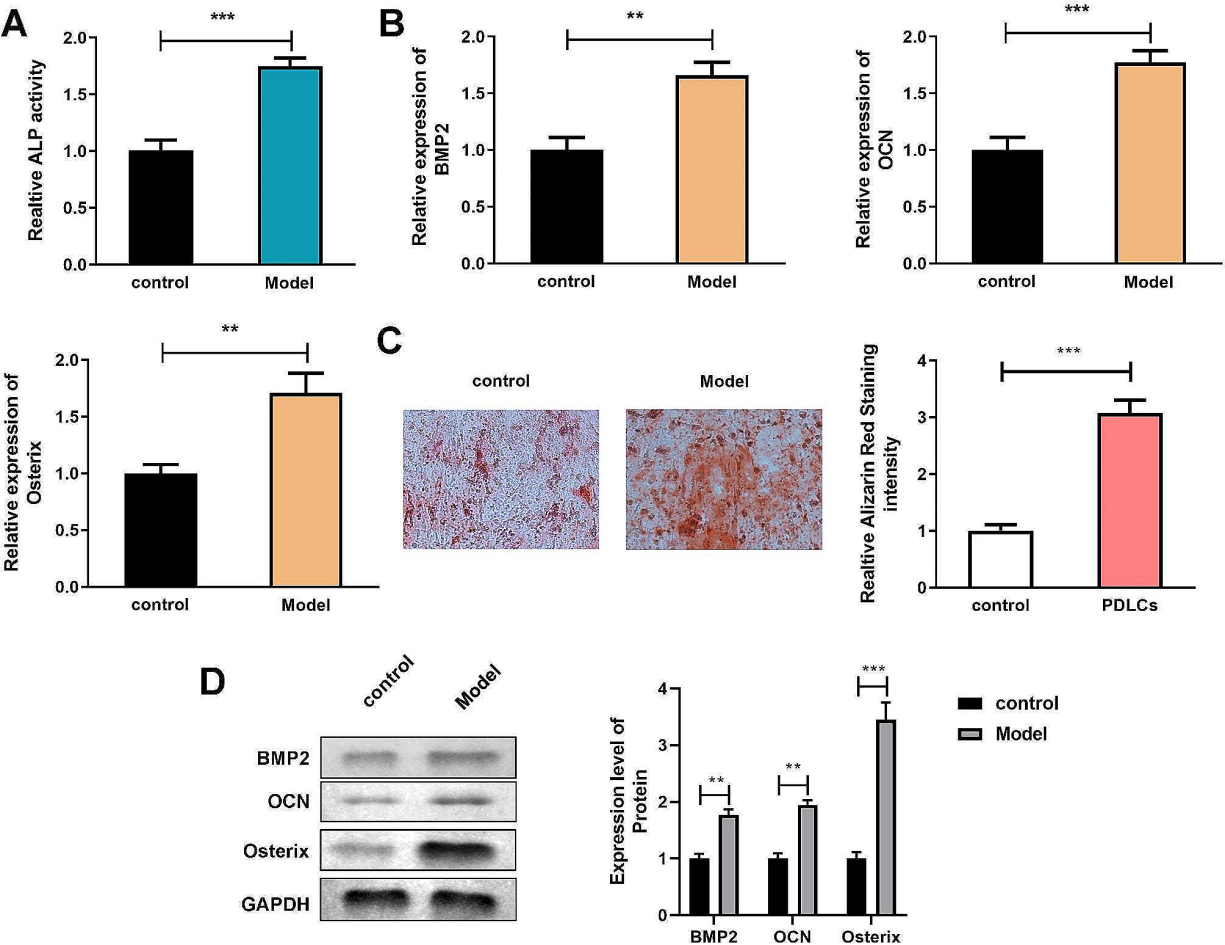
Differentiated hMSCs reveals the osteoblast differentiation capacity. ALP activity of hMSCs were detected by ALP activity assay. B. mRNA expression levels of BMP2, OCN, and Osterix were measured by qRT-PCR. C. Calcium mineralized level of hMSCs were detected by ARS staining. D. Protein expression levels of BMP2, OCN, and Osterix were measured by western blot assay. **P < 0.01, ***P < 0.001

ALP activity assay and ARS staining was applied to determine the level of osteoblast differentiation. Differentiated hMSCs showed a high ALP activity compared with hMSCs before osteoblast differentiation induction (Fig. 2A). Meanwhile, the mineralization nodules stained by ARS in differentiated hMSCs were notably more than control group (Fig. 2C). Then, the expression of osteogenic-related proteins including BMP2, OCN, and Osterix were detected to further show the ability of osteoblast differentiation of differentiated hMSCs. The results indicated that both mRNA and protein expression levels were increased after hMSCs were differentiated (Fig. 2B and D).

### ULBP1 impaires osteoblast differentiation

**Fig. 3:**
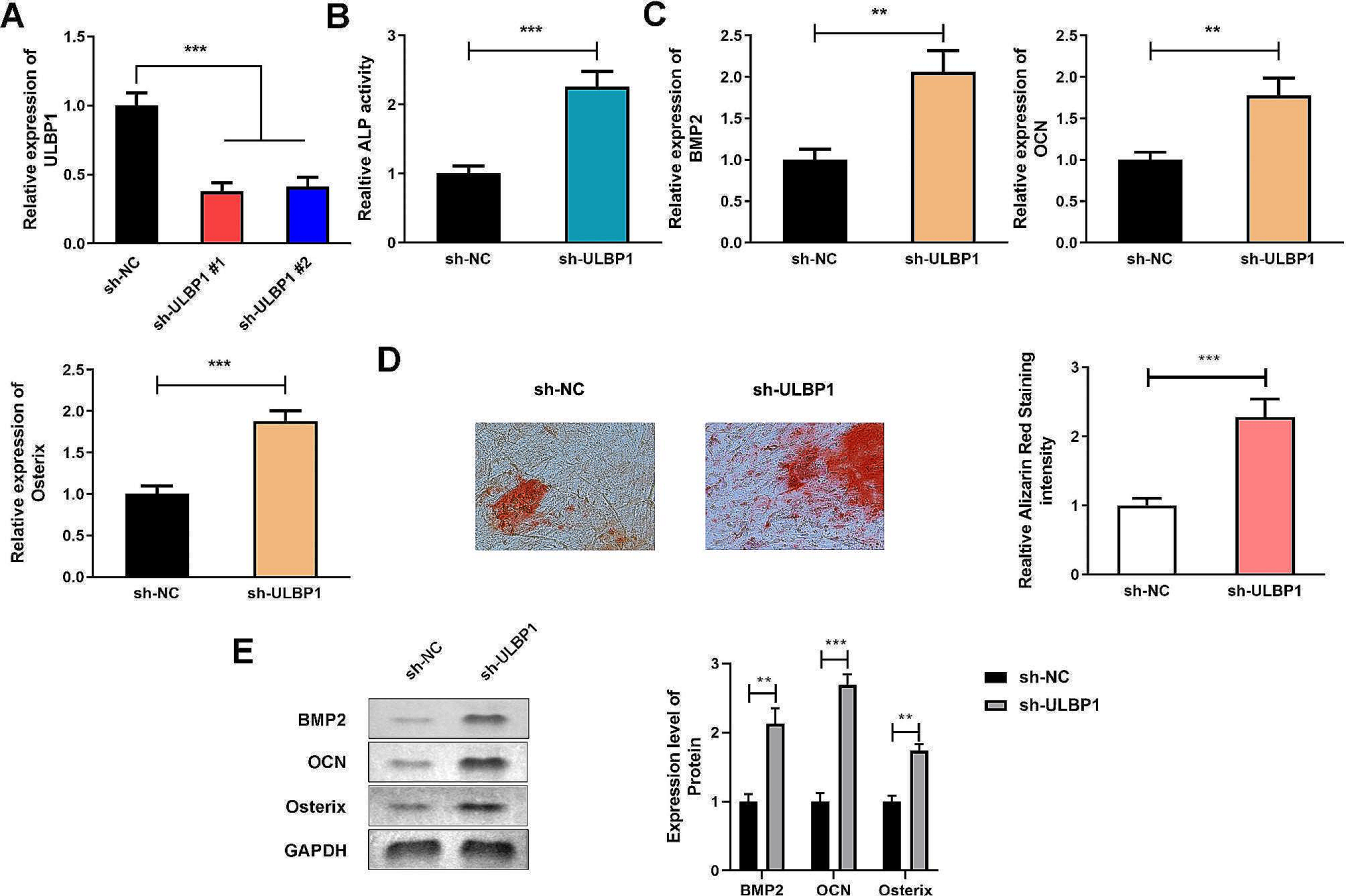
ULBP1 impaires osteoblast differentiation. ULBP1expression levels were detected by qRT-PCR after transfected. B. ALP activity of hMSCs were detected by ALP activity assay. C. mRNA expression levels of BMP2, OCN, and Osterix were measured by qRT-PCR. D. Calcium mineralized level of hMSCs were detected by ARS staining. E. Protein expression levels of BMP2, OCN, and Osterix were measured by western blot assay. **P < 0.01, ***P < 0.001

As shown in Fig. 3A, mRNA expression of ULBP1 was suppressed after transfected with two designed shRNAs. ALP activity as well as calcium mineralized level of differentiated hMSCs were significantly increased after ULBP1 was knockdown (Fig. 3B and D). Likewise, BMP2, OCN, and Osterix expression levels were also up-regulated induced by suppressed ULBP1 (Fig. 3C and E). The elevated ULBPL1, however, exerted the opposite way on regulating ALP activity, ARS staining intensity, OCN, and Osterix expression levels (Fig. S2).

### ULBP1 related genes are enriched in TNF-β signaling pathway

**Fig. 4:**
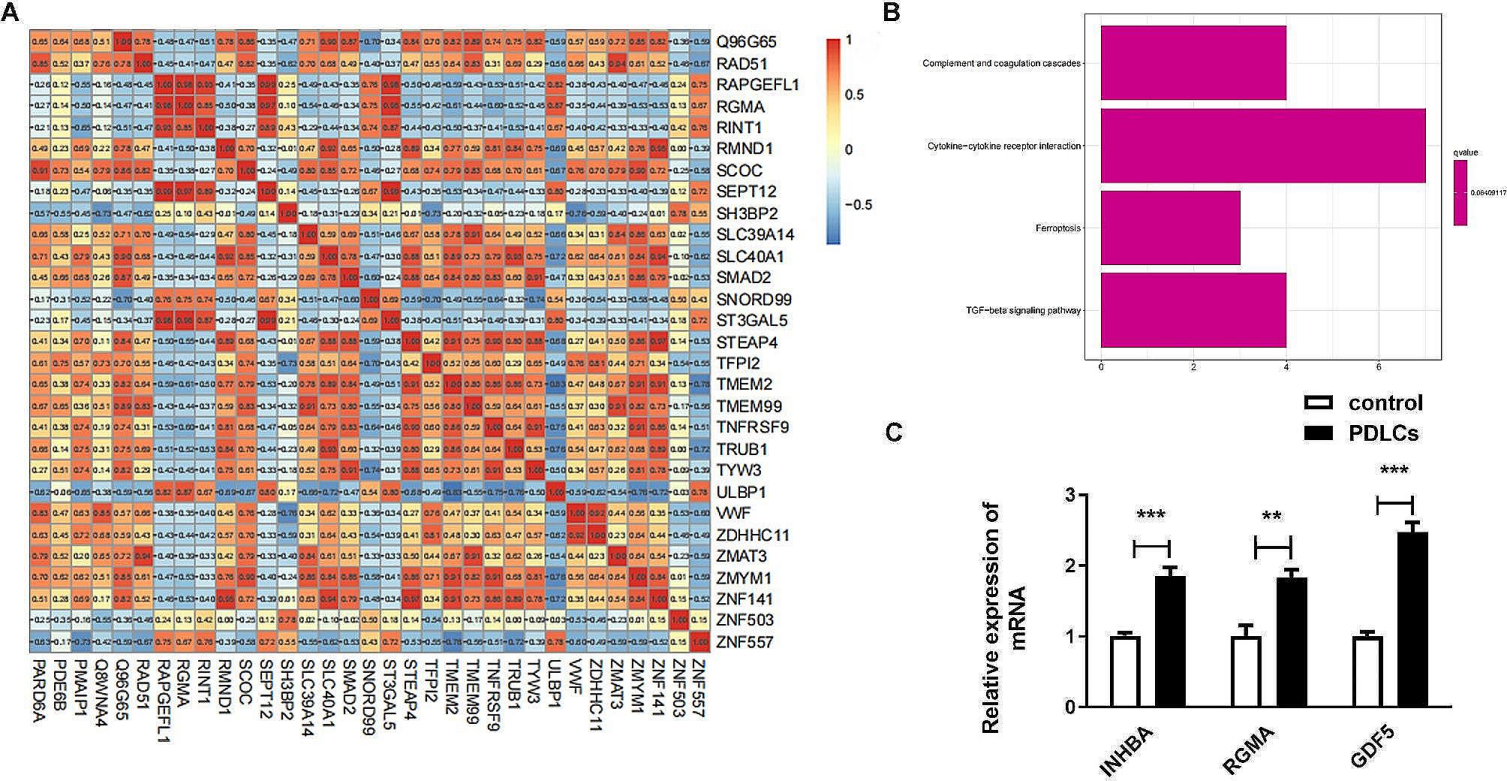
ULBP1 related genes are enriched in TNF-β signaling pathway. Pearson correlation analysis between ULBP1 gene and other DEGs. B. KEGG analysis of DEGs inversely associated with ULBP1 gene. C. Expression levels of INHBA, RGMA, and GDF5 enriched in TGF-β signaling pathway. **P < 0.01, ***P < 0.001

The correlation between ULBP1 gene and other DEGs identified was conducted by Pearson correlation analysis (Fig. 4A). Then, the results of KEGG analysis indicated that DEGs inversely associated with ULBP1 gene were enriched in four pathways including complement and coagulation cascades, cytokine-cytokine receptor interaction, ferroptosis, and TGF-β signaling pathway (Fig. 4B). INHBA, RGMA, and GDF5 are three DEGs enriched in TGF-β signaling pathway, and were negatively related to ULBP1. Moreover, in the differentiated hMSCs, expression levels of three DEGs enriched in TGF-β signaling pathway in differentiated hMSCs were all up-regulated (Fig. 4C).

### Suppressed ULBP1 promotes osteoblast differentiation by activating TNF-β signaling pathway

**Fig. 5:**
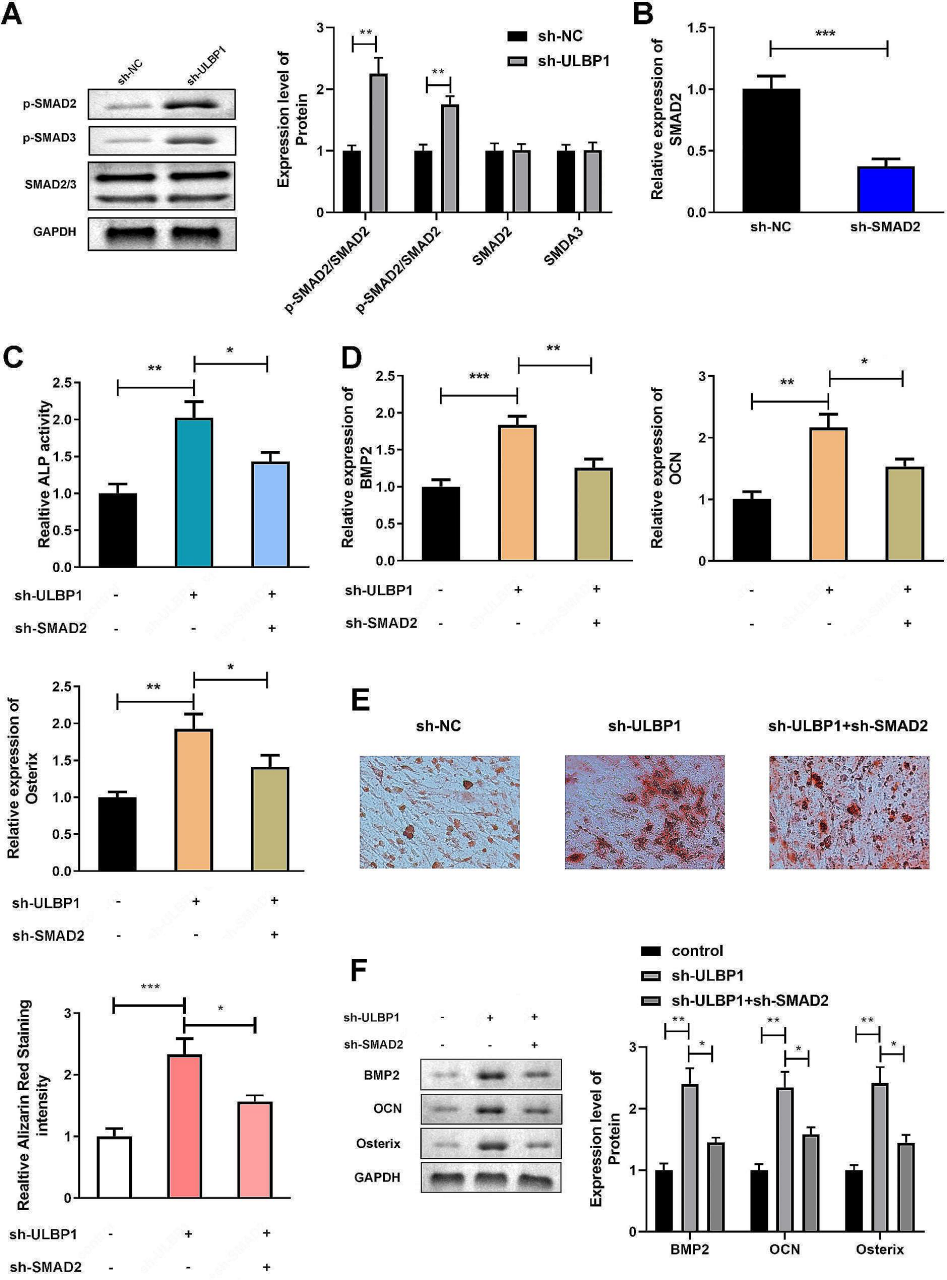
Suppressed ULBP1 promotes osteoblast differentiation by activating TNF-β signaling pathway. Protein expression of SMAD2/3 as well as phosphorylated SMAD2/3 were measured by western blot assay. B. SMAD2 expression levels were detected by qRT-PCR. C. ALP activity of hMSCs were detected by ALP activity assay. D. mRNA expression levels of BMP2, OCN, and Osterix were measured by qRT-PCR. E. Calcium mineralized level of hMSCs were detected by ARS staining. F. Protein expression levels of BMP2, OCN, and Osterix were measured by western blot assay. *P < 0.05, **P < 0.01, ***P < 0.001

Figure 5A indicated that protein expression of SMAD2/3 as well as phosphorylated SMAD2/3 were dramatically increased after suppression of ULBP1 gene in differentiated hMSCs. Then, SMAD2 was knockdown to evaluate the effect of suppressed ULBP1 on osteoblast differentiation capacity (Fig. 5B). Increase of ALP activity as well as calcium mineralized level of differentiated hMSCs induced by silencing ULBP1 were obviously attenuated by deficiency of SMAD2, as did the expression of BMP2, OCN, and Osterix (Fig. 5C and F).

### ULBP1 knockdown decreases bone loss induced by ovariectomy

**Fig. 6:**
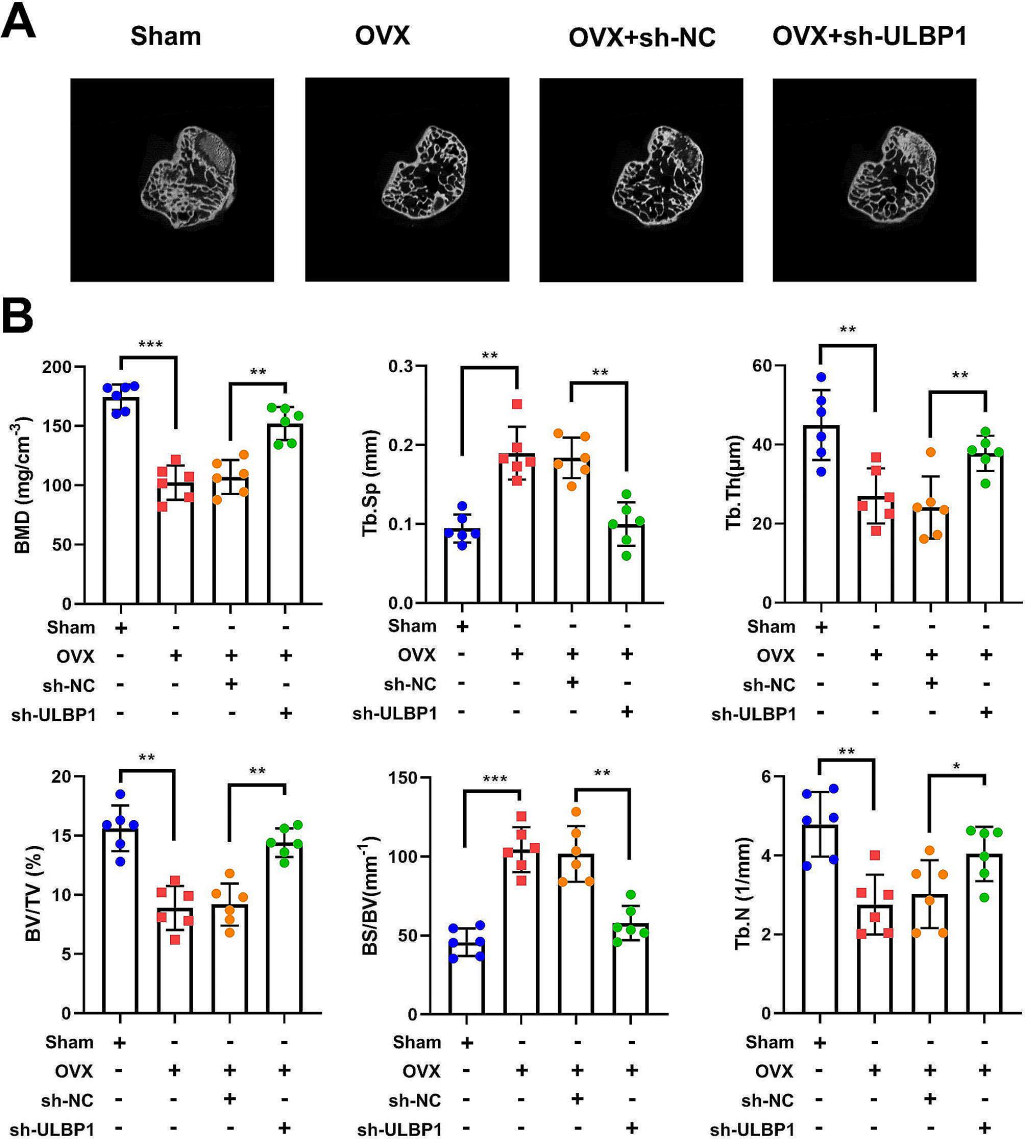
ULBP1 knockdown decreases bone loss induced by ovariectomy. Representative micro-CT images of trabecular bone from the femoral metaphysis in different mice group. B. Quantification of micro-computed tomography data including BMD, Tb.Sp, Tb. Th, BV/TV, BS/BV, and Tb. N. **P < 0.01, ***P < 0.001

To evaluate the potential role of *ULBP1* in bone loss induced by ovariectomy, ovariectomized 10-week-old mice were treated with an intravenous injection of *ULBP1* knockdown lentivirus or control lentivirus starting 3 days after ovariectomy. Micro-CT analysis revealed that *ULBP1* knockdown decreased ovariectomy-induced bone loss (Fig. 6A). The quantitative analysis revealed that differences in BMD, Tb.Sp, Tb.Th, BV/TV, BS/BV, and Tb.N in trabecular bone were all significant between sham and OVX groups (Fig. 6B). These data revealed that OVX surgery prominently attenuated bone microstructure and bone mass; however, knockdown of *ULBP1* dramatically reversed the effects of OVX treatment.

## Discussion

Osteoporosis is a multifactorial disease with genetic and strong epigenetic components [4]. Although there is a large number of candidate genes association studies [16, 17], the etiology and molecular mechanisms of the disease are not fully understood. In this study, ULBP1 gene has been identified to be overexpressed in osteoporosis patients whereas was down-regulated in differentiated hMSCs. Suppression of ULBP1 promoted osteoblast differentiation capacity via activating TNF-β signaling pathway.

ULBP1 is one of the ligands of natural killer group 2 member D (NKG2D) and mediates natural killer (NK) cell cytotoxicity [12, 13], and has been identified to be a DEGs in osteoporosis. Overexpressed ULBP1 could activate NK cells to regulate disorders including tumors and preeclampsia [18, 19]. However, the effects of ULBP1 in osteoporosis remain unreported. Interestingly, a sub-type of NK cells were reported to function as a regulator in maintaining bone homeostasis in osteoporosis [20, 21]. Hence, we speculated that aberrant expressed ULBP1 negatively related to NK cells may play a regulatory role in maintaining balance between osteoblasts and osteoclasts. Our data suggested that ULBP1 presented to be upregulated in serum of osteoporosis, while downregulated in hMSCs with higher osteoblast differentiation. Then, ULBP1 suppression decreased capacity of osteoblast differentiation in hMSCs, indicating that ULBP1 may inhibit osteoblast differentiation.

Transforming growth factor-β (TGF-β) signaling pathway plays an important role in maintaining bone homeostasis [22, 23]. Phosphorylation of TGF-β receptors has the ability to recruit and phosphatize serine sites at the the C-terminal of SMAD2/3 [24, 25]. TGF-β activates classical SMAD-dependent signaling pathway to modulate related transcription factors to regulate osteogenic and adipogenic differentiation of mesenchymal stem cells [26]. For instance, Activated TGF-β1/SMADs signaling pathway induced by abnormally expressed miR-497 or LRG1 promoted osteoblastic activity and collagen synthesis [27]. Likewise, MOTS-c activated TGF-β/SMAD pathway to promoted the synthesis of type I collagen in osteoblasts, thereby improving osteoporosis [28]. In this research, results of pearson analysis along with KEGG analysis of DGEs demonstrated that ULBP1 could suppressed osteoblast differentiation by inactivating TNF-β signaling pathway, which was in line with previous studies [27, 28]. Furthermore, suppressed TNF-β signaling pathway induced by inactivating SMAD2 significantly attentuated osteoblast differentiation promoted by deficiency of ULBP1.

There are some limitations in this study. The ULBP1 gene has been identified in the osteoclast precursor cells sorted from the circulating monocytes of patients with osteoporosis and normal controls according to the bioinformatic analysis. The role of ULBP1 gene in regulating the osteoclastic differentiation should be studied in the future.

## Conclusion

Hence, ULBP1 suppression promoted osteoblast differentiation of hMSCs via activating TNF-β signaling pathway. Knockdown of ULBP1 may be an alternative for the treatment of osteoporosis.

### Electronic supplementary material

Below is the link to the electronic supplementary material.

Supplementary Material 1

Supplementary Material 2

Supplementary Material 3

Supplementary Material 4

## Data Availability

The datasets used and/or analyzed during the current study are available from the corresponding author on reasonable request.
