# Supplementary material for: Knockdown of the UL-16 binding protein 1 promotes osteoblast differentiation of human mesenchymal stem cells by activating the SMAD2/3 pathway
Source: BMC Musculoskelet Disord. 2024 Mar 13;25:213. doi: 10.1186/s12891-024-07341-0 (PMC10936096; doi:10.1186/s12891-024-07341-0)
Supplement: Supplementary file 1 — Supplementary Material 1 [file 12891_2024_7341_MOESM1_ESM.docx]

**Materials and methods**

**Overexpression of ULBP1**

For stable transfection, lentiviruses carrying target gene overexpression sequence or corresponding controls were purchased from Genechem (Shanghai, China). Then transfected into the differentiated hMSCs according to manufacturer's instructions and selected with puromycin for about 7 days.

**ELISA**

The protein levels of ULBP1 in serum samples collected from patients with osteoporosis and controls were evaluated by a human ULBP1 ELISA kit (ab267647) from Abcam (CA, USA).

**Supplementary Figure Legends**

**Figure S1. ULBP1 protein levels in serum samples of patients with osteoporosis and healthy controls measured by ELISA**

**Figure S2.** ***ULBP1* impaires osteoblast differentiation.**

1. ULBP1expression levels were detected by qRT-PCR after transfected. B. ALP activity of hMSCs were detected by ALP activity assay. C. mRNA expression levels of BMP2, OCN, and Osterix were measured by qRT-PCR. D. Calcium mineralized level of hMSCs were detected by ARS staining. E. Protein expression levels of BMP2, OCN, and Osterix were measured by western blot assay.

***P*<0.01, ****P*<0.001.
