# Supplementary figures and images for: Knockdown of the UL-16 binding protein 1 promotes osteoblast differentiation of human mesenchymal stem cells by activating the SMAD2/3 pathway
Source: BMC Musculoskelet Disord. 2024 Mar 13;25:213. doi: 10.1186/s12891-024-07341-0 (PMC10936096; doi:10.1186/s12891-024-07341-0)

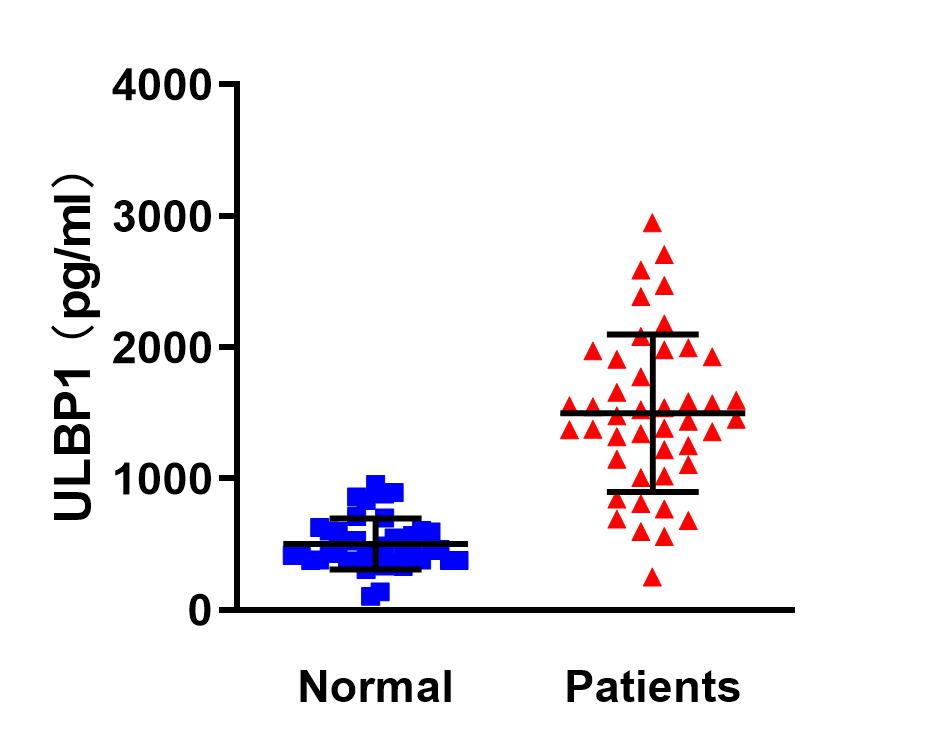

Supplement: Supplementary file 2 — Supplementary Material 2 [file 12891_2024_7341_MOESM2_ESM.jpg]

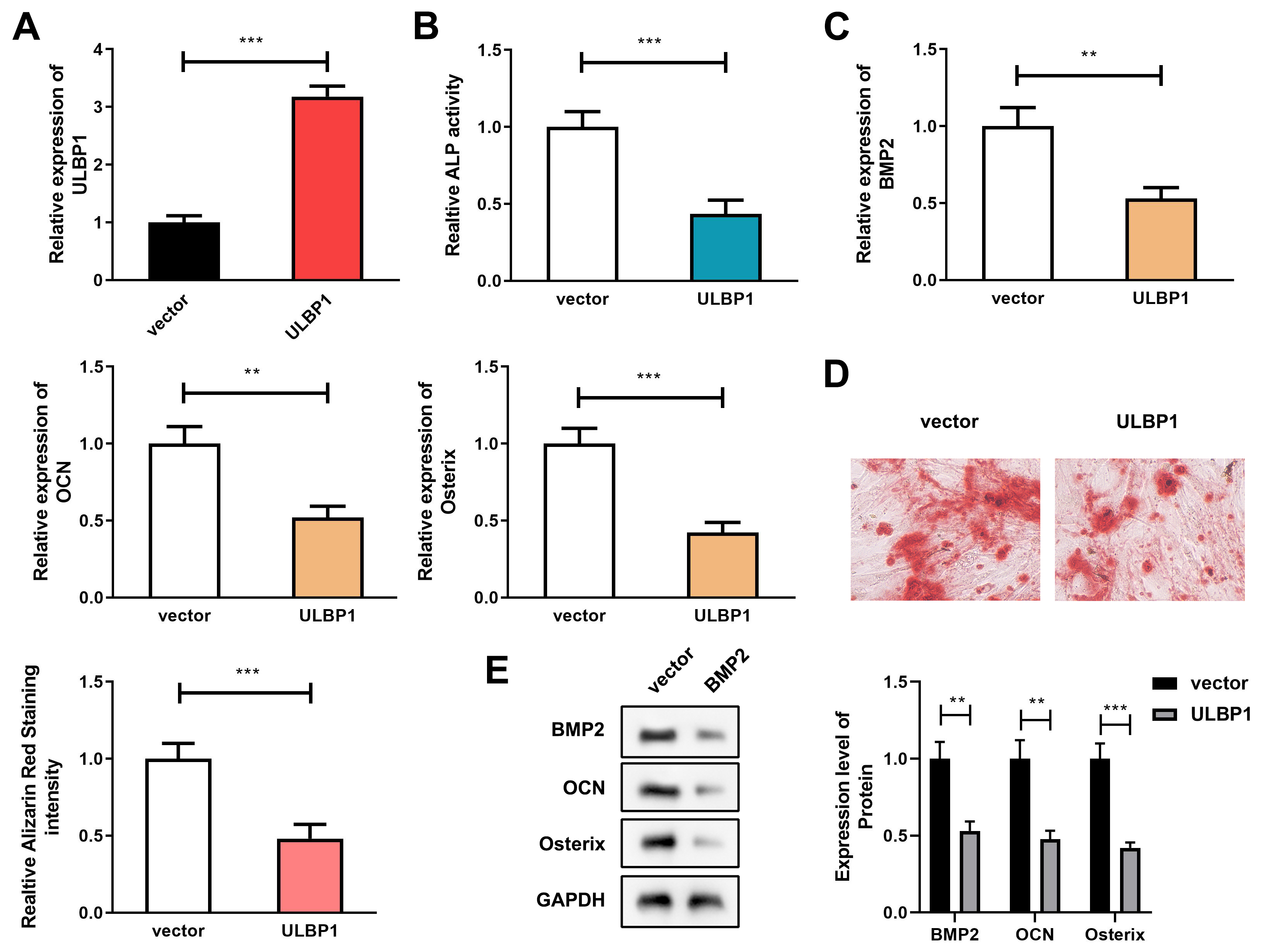

Supplement: Supplementary file 3 — Supplementary Material 3 [file 12891_2024_7341_MOESM3_ESM.jpg]

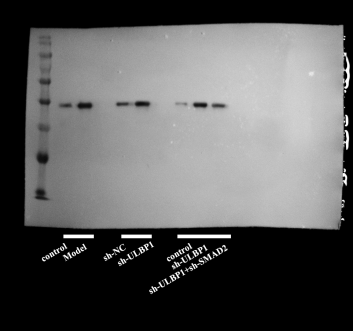


BMP2


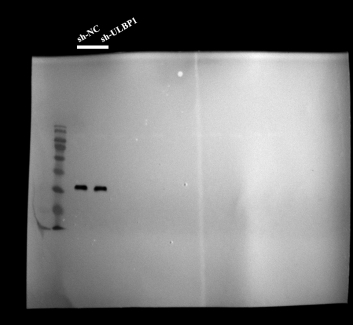


GAPDH (2)


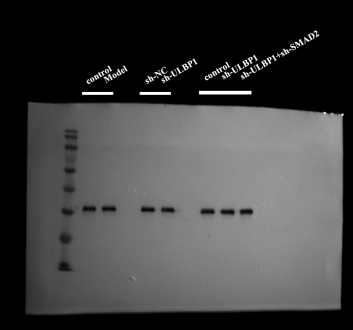


GAPDH


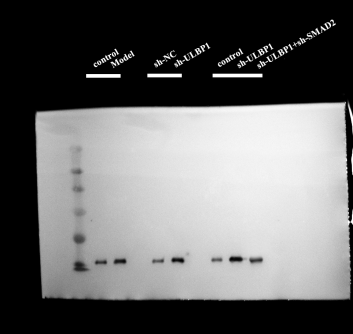


OCN


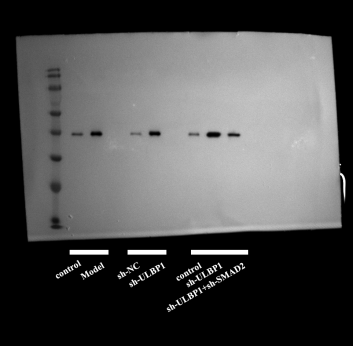


OSTERIX


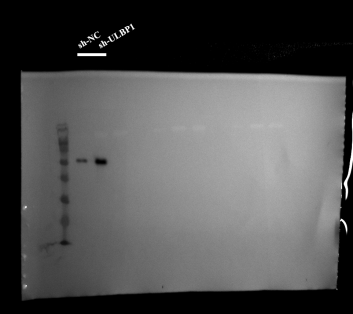


p-SMAD2


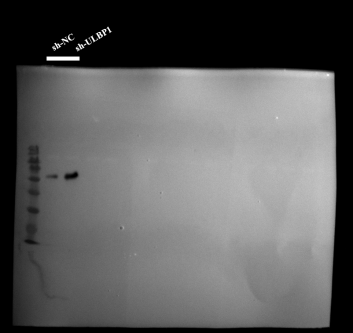


p-SMAD3


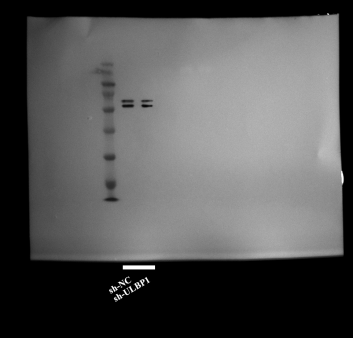


SMAD23

**Fig. S2**


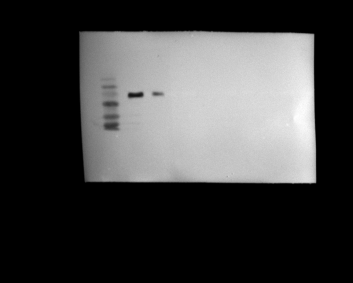


BMP2


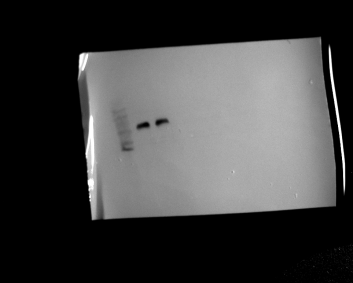


GAPDH


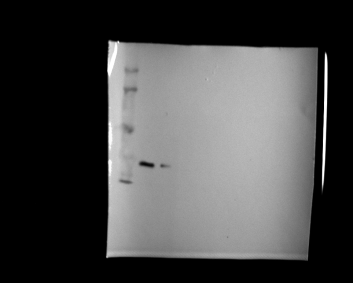


OCN


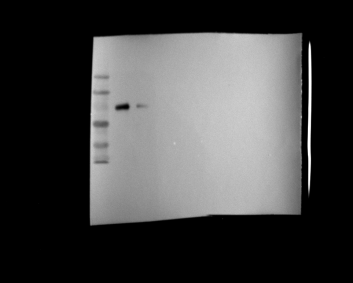


OSterix

Supplement: Supplementary file 4 — Supplementary Material 4 [file 12891_2024_7341_MOESM4_ESM.docx]
